# Ferromagnetic excess moments and apparent exchange bias in FeF_2_ single crystals

**DOI:** 10.1038/s41598-019-55142-6

**Published:** 2019-12-11

**Authors:** D. C. Joshi, P. Nordblad, R. Mathieu

**Affiliations:** Department of Engineering Sciences, Uppsala University, Box 534, SE-751 21 Uppsala, Sweden

**Keywords:** Magnetic properties and materials, Magnetic properties and materials

## Abstract

The anisotropic antiferromagnet FeF_2_ has been extensively used as an antiferromagnetic layer to induce exchange bias effects in ferromagnetic/antiferromagnetic bilayers and heterostructures. In this work, an apparent exchange bias occurring in the low temperature hysteresis loops of FeF_2_ single crystals is investigated. A detailed investigation of the hysteresis and remnant magnetization indicates that the observation of an apparent exchange bias in FeF_2_ stems from an intrinsic excess moment associated with a distortion of the antiferromagnetic structure of piezomagnetic origin.

## Introduction

The iron fluoride FeF_2_ has been extensively used as an antiferromagnetic layer to induce exchange bias effects in ferromagnetic/antiferromagnetic bilayers and heterostructures, a phenomenon of large interest in technological applications such as magnetoresistive read heads and spintronics^1^. The strongly anisotropic uniaxial antiferromagnet FeF_2_ is an almost ideal realization of a 3d-Ising model system. It exhibits an antiferro- to paramagnetic transition across the Néel temperature T_N_ = 78.4 K^2^. Analyzes of the critical behavior of the specific heat, derived from direct measurements^2^ and the derivative of the temperature dependence of the linear birefringence d(Δn)/dT, yields a critical exponent α = 0.11^3^ in agreement with analytic theory^4^. The system is a highly anisotropic material with an anisotropy energy hundred times larger as compared to MnF_2_, another family member of 3*d*- metal fluorides^5,6^. Internal random stress in such a system may generate a weak spontaneous magnetic moment, piezomagnetic response, due to unbalanced magnetic moments in the two sub-lattices^7^. Previous studies of the temperature dependence of the excess moment shows that it gradually decreases with increasing temperature and approaches zero at T_N_^8^. In the antiferromagnetic state, the spontaneous moment is locked in the cooling field direction and becomes independent of the applied magnetic field and in the transition region, this moment is proportional to the staggered magnetization m_s_ (M ∝ m_s_ ∝ (T-T_N_)^β^) yielding a value of the critical exponent β = 0.325^8^ in agreement with the theoretical 3d-Ising model value^3^. Diluting the antiferromagnet FeF_2_ with non-magnetic ions such as Zn (Fe_*x*_Zn_1−*x*_F_2_) provides a system which in a homogenous magnetic field (Dilute Antiferromagnet in a uniform magnetic field (DAFF)) is a physical realization of the random field Ising model^9,10^. In Fe_0.46_Zn_0.54_F_2_, the hysteresis (M-H) curves were found to include an excess moment under field cooled (FC) conditions, somewhat similar to that observed in dilute magnetic alloys such as Ni(Mn), Cu(Mn), Ag(Mn), where a unidirectional anisotropy leads to horizontally shifted hysteresis loops, reminiscent of exchange bias effects^11–15^. The intercalated transition metal dichalcogenide Fe_*x*_NbS_2_ is another compound that shows a peculiar exchange bias (EB) phenomenon, which by Doyle *et al*.^16^ was attributed to the coexistence of spin glass and long-range antiferromagnetic ordering.

In this study, we show that an apparent exchange bias observed in the low temperature hysteresis loops of single crystals of FeF_2_ originates from a vertical shift of the loops due to an intrinsic excess moment associated with a distortion of the antiferromagnetic structure appearing when passing through T_N_ in a finite magnetic field.

## Results and Discussion

**Figure 1:**
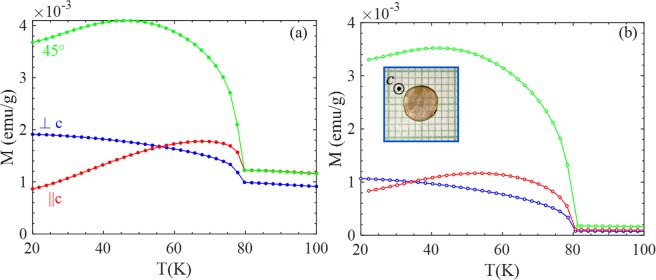
Temperature dependence of (**a**) magnetization M under FC in a magnetic field of H = 5 Oe, and (**b**) thermo-remnant magnetization (TRM) measured along perpendicular (⊥c), 45° and parallel to *c*-axis (||c) of the FeF_2_ circular disc. The inset shows a photograph of the top view (along the c-axis) of the FeF_2_ single crystal used in experiment.

**Figure 2:**
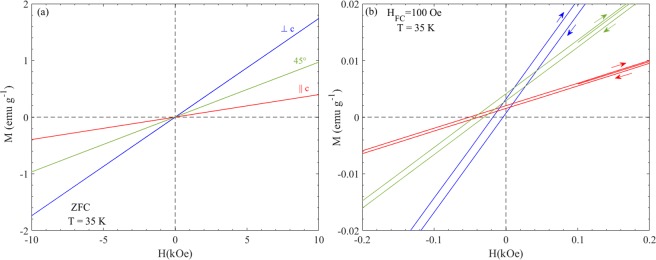
M(H) recorded with (**a**) field sweep 0 → +1 T → −1 T → +1 T after ZFC from 100 K down to 35 K, for three different orientations. (**b**) M(H) recorded with field sweep 100 Oe → +1 T → −1 T → + 1 T after FC in 100 Oe from 100 K down to 35 K, same color legend as in (**a**).

Figure 1 shows the temperature dependence of (a) field cooled (FC); and (b) thermo-remnant magnetization (TRM) curves measured under a constant magnetic field H = 5 Oe for three different orientations of FeF_2_ circular disk; (*i*) with H ⊥ c-axis (blue color), (*ii*) H ∠45° (green color) and (*iii*) H || c-axis (red color). The FC and TRM curves exhibit a sharp upturn across the magnetic ordering temperature T_N_ ~79 K, associated with the unbalanced magnetic moment of the two sub-lattices. The T_N_ determined from the M(T) measurement is consistent with the value of T_N_ determined from the specific heat data reported by Chirwa *et al*.^2^. The TRM values ~1.1 × 10^−3^ emu/g (1.8 × 10^−5^ μ_B_/Fe-atom) and 0.8 × 10^−5^ emu/g (1.4 × 10^−5^ μ_B_/Fe-atom) at T = 20 K and H = 5 Oe for ⊥c and ||c configuration are also of similar magnitude as earlier reported values of the excess moment^2,8^; Fig. SM1 in the supplementary material shows the zero field cooled (ZFC) as well as TRM curves in μ_B_/Fe-atom for reference. Below T_N_ an irreversibility between the ZFC and FC curves associated with the uncompensated magnetic moments occurs. At higher fields the AFM susceptibility dominates over the FM excess moment and yields a typical AFM M(T) curve for fields parallel to the c-axis, as shown in Supplementary Fig. SM2 for the ||c configuration. The combination of AFM susceptibility and excess moment imposes an apparent exchange bias in this system. Figure 2(a) shows the magnetic hysteresis loops (M(H) loops) recorded at T = 35 K with field sweep 0 → +1 T → −1 T → +1 T after ZFC for ⊥c (blue color), ∠45° (green color) and ||c (red color) orientations of FeF_2_ single crystal. An apparent exchange bias, shift in the hysteresis loops, is observed for all the three different orientations albeit, as we discussed below this shift is dependent on the cooling and measurement conditions. This shift is more clearly defined when M(H) is recorded after FC with H_FC_ = 100 Oe at T = 35 K as shown in Fig. 2(b). The observed shift −H_EB_ is 10 Oe, 37 Oe and 40 Oe for ⊥c, ∠45° and ||c orientations, respectively. This apparent shift in M(H) curves disappear when the curves are recorded at T = 100 K (T > T_N_) as shown in Supplementary Fig. SM3, confirming the coupling between AFM order and FM excess moment below T_N_. A shift in the M(H) loops towards opposite direction (right hand side) was noticed when the sample is cooled in a negative field (−H_FC_). The systematic variation of the shift in M(H) curves when cooled in various positive and negative fields (±H_FC_) and recorded at T = 35 K for three different orientations are shown in Figs. SM4–,6. Apart from the apparent exchange bias, there is some irreversibility (horizontal shifts) in the M(H) loops (inset *i* of Fig. 2a). This ‘coercivity’ is an artifact associated with the field-history dependence of the remnant field of the superconducting magnet in the MPMS system^16^, which causes a difference between the read off field measure (proportional to the current through the superconducting magnet) and the actual field at the sample. Although, before performing all these measurements we have used the ultra-low field option, a stray/remnant field appears after applying magnetic fields H > 1 kOe. As seen in Fig. SM7, the initial value of the magnetization in M(H) measurements recorded after ZFC depends on the weak cooling field remaining in the system. If we limit the maximum field to 1 kOe in M(H) measurements (−1 kOe ≤ H ≤ +1 kOe) the irreversibility vanishes as shown in the inset of Fig. SM7, confirming the artificial origin of coercivity due to stray fields.

**Figure 3:**
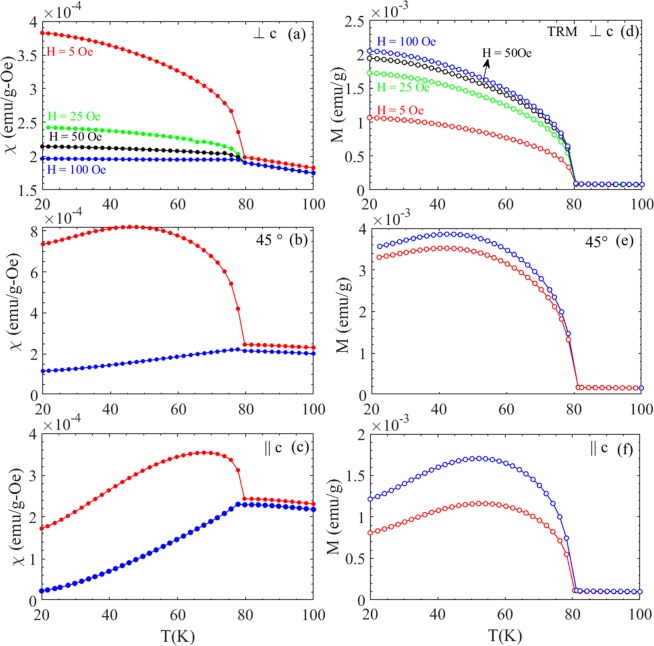
(left) FC magnetization plotted as M/H and (right) the TRM magnetization for two fields (H = 5 and 100 Oe) and three different orientation (**a**) and (**d**) ⊥c, (**b**) and (**e**) 45° and, (**c**) and (**f**) parallel to *c*-axis (||c); in the case of ||c configuration data for H = 25 and 50 Oe is added. (See Fig. SM1 for SI units and corresponding µ_B_/Fe value).

Figure 3 displays the temperature dependence of the magnetic susceptibility χ(T) (left panel) and the TRM (right panel) recorded at two different magnetic fields H = 5 Oe and 100 Oe, for ⊥c, ∠45° and ||c orientations. For ||c orientations, data for H = 25 Oe and 50 Oe is also added. The representation of χ(T) in SI units for both ZFC and FC magnetization, and M_TRM_(T) in µ_B_/Fe are shown for reference in Supplementary Fig. SM1. The weak ferromagnetism at low field is attributed to an excess moment locked to the direction of the cooling field. The susceptibility in the H ∠45° case lies in-between the susceptibilities of ⊥c and ||c oriented samples as seen from the M(H) curves shown in Fig. 2(a). However, the remnant/spontaneous magnetization for H ∠45° is nearly three times larger than in the ||c case and twice greater than for H ⊥ c, as can be noticed from the M(H) curves in Fig. 2 near H = 0 or the TRM curves in Fig. 3e. This behavior may relate to the theoretical observation for some other metal fluorides where the spontaneous piezomagnetic moment has a preferred crystallographic orientation, which is different that the main crystallographic axes^7,17^.

**Figure 4:**
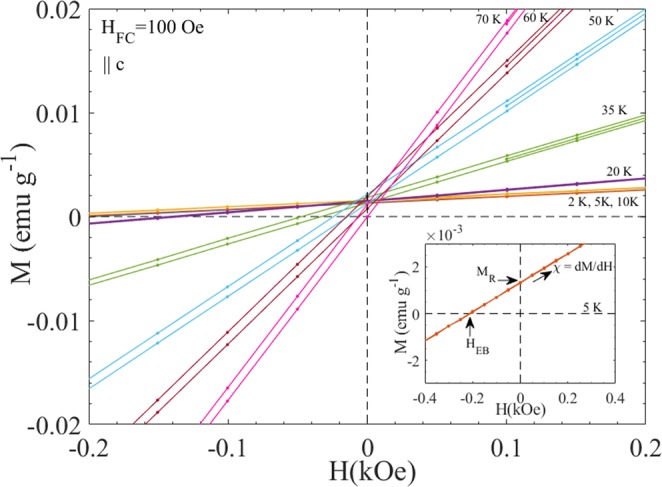
M(H) recorded with field sweep 100 Oe → +1 T → −1 T → +1 T after FC in 100 Oe from 100 K down to low temperature for ||c orientation of FeF_2_ circular disk. Inset shows the M(H) curve at T = 5 K of main panel plotted within the magnetic field range of −0.4 kOe ≤ H ≤ +0.4 kOe.

**Figure 5:**
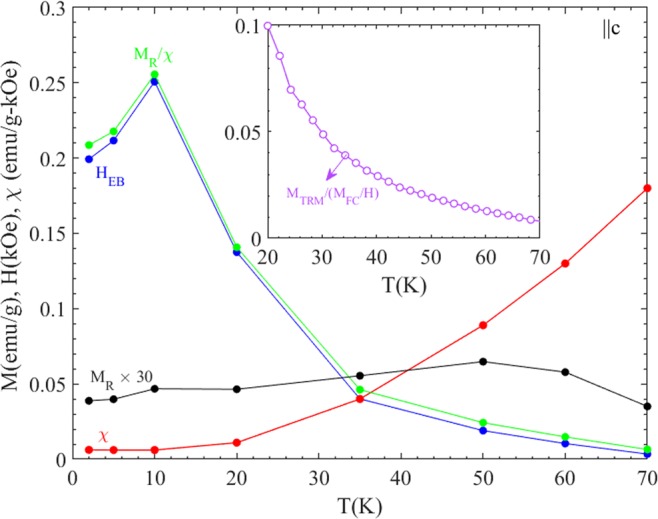
Temperature dependence of H_EB_ (blue color) determined from Fig. 4 for ||c orientation. For comparison M_R_ (scaled by a factor of 30 for clear visibility), χ = dM/dH and M_R_/χ (green color) extracted from the same M(H) data are also plotted on the same axis. Inset shows the temperature dependence of the ratio of M_TRM_ at 100 Oe (Fig. 3f) and susceptibility M_FC_/H recorded at H = 1 T (Supplementary Fig. 2) for comparison (||c orientation) with H_EB_.

Figure 4 shows the magnetic hysteresis loops (M(H) loops) after FC in H_FC_ = 100 Oe from 100 K down to low temperatures (2 K ≤ T_m_ ≤ 70 K) for ||c orientation. The M(H) data after cooling in H_FC_ = 25 Oe and recorded at three different temperatures T = 50 K, 35 K and 10 K are shown in Supplementary Fig. SM8 for comparison. As T_m_ is decreased from 70 K to 2 K, a significant shift in the hysteresis loop towards left hand side as well as decrease in the slope of M(H) curve is observed. To clearly visualize the shift and change in slope (susceptibility), the M(H) curve at T = 5 K of the main panel is plotted within the magnetic field range of −0.4 kOe ≤ H ≤ +0.4 kOe in the inset. Here the horizontal and vertical shifts are identified as −H_EB_(H_EB_ > 0) and M_R_ respectively; the high field slope of the M(H) curve, dM/dH = χ is also derived. Figure 5 shows the temperature dependence of −H_EB,_ M_R_ (scaled by a factor of 30 for clear visibility), χ and M_R_/χ extracted from the M(H) data in Fig. 4. The inset shows the temperature dependence of the ratio of M_TRM_ at 100 Oe (Fig. 3f) and high-field susceptibility M_FC_/H recorded at H = 1 T (Fig. SM2) (in the ||c orientation). The close covariation of −H_EB_(T) and M_R_/χ(T) demonstrates that the apparent exchange bias observed in FeF_2_ stems from its excess moment (M_R_) and its interplay with the antiferromagnetic susceptibility (χ). As shown in Fig. SM9, the linear shape of the M(H) loops remains even if larger fields (9 T) are applied in the measurements.

## Conclusion

An intrinsic excess moment is induced in the antiferromagnetic structure of FeF_2_ when cooling through the Néel temperature in a finite magnetic field. This moment is confined to the direction of the cooling field and saturates already at cooling fields of order 100 Oe. The magnitude of the excess moment is strongly directional dependent and largest when the cooling field is applied at an angle with respect to the c- and a-axes. The excess moment gives rise to tunable apparent exchange bias due to vertically shifted hysteresis loops. The measured exchange bias becomes large at low temperatures with the applied field along the crystallographic c-axis, since the parallel susceptibility of FeF_2_ approaches zero at low temperatures. An apparent coercivity of the high field hysteresis loops measured in MPMS systems (or PPMS systems) is caused by the history dependent remanent field in the superconducting magnet of the magnetometer. The finding that the excess moment has its largest amplitude at an angle with respect to the main crystallographic axes is consistent with previous results from studies of piezomagnetic effects for some other metal fluorides. Since the works by Dzialoshinskii^17^, relatively few theoretical studies have been undertaken to account for these specific effects. There are some studies, which aimed to probe the structure and magnetic properties relationship using pressure in those materials, and we hope that our study stimulates new experimental^18^ and theoretical studies^6^ of piezomagnetism.

## Methods

The temperature and field dependent magnetization measurements for three different orientations of FeF_2_ circular disk (mass 104.56 mg; 5 mm diameter/1.25 mm thick) were performed by using a superconducting quantum interference device (SQUID) magnetometer from Quantum Design Inc (Model:XL) equipped with the ultralow field option. The magnetic field H was applied in the plane of the disk (perpendicular to c axis of the structure, denoted ⊥c), perpendicular to the plane (parallel to c; denoted ||c) and with a 45 degree tilt angle with respect to the plane of the disk (45 degree tilted disk, denoted 45°). The dependence of the magnetization M on the temperature T was recorded in zero-field cooled (ZFC) and field cooled (FC) conditions under magnetic fields H of 5 Oe, 25 Oe, 50 Oe and 100 Oe. For ZFC measurement, the sample was cooled to low temperature (T < T_N_) under zero field, and then the sample was subjected to a constant dc-magnetic field H before recording the data during warming the sample. The FC data was recorded during a subsequent cooling in the same magnetic field. The thermo-remnant magnetization (TRM) was recorded on warming in zero magnetic field, after cooling the sample from 100 K down to 20 K in presence of a constant magnetic field. The field dependence of magnetization M(H) was recorded at a temperature T after zero-field cooling from 100 K down to T_m_; with field swept from 0 → +1 T → −1 T → +1 T (ZFC), The M(H) were also recorded under field cooled conditions with field swept from + H_FC_ → +1 T → −1 T → +1 T after field cooling in H_FC_ (H_FC_ > 0) from 100 K down to T_m_ (FC). Measurements with field swept from 0 → −1 T → +1 T → −1 T (ZFC) and −H_FC_ → −1 T → +1 T → −1 T after field cooling in −H_FC_ (−H_FC_ < 0) from 100 K down to T_m_ (FC) were also performed for comparison. Before performing all of these measurements, the magnetic field of the magnet was reset to zero by using the ultra-low field option. A Physical property measurement system (PPMS) with VSM option from Quantum Design was used to record M(H) curves up to high fields H = 9 T.

## Supplementary information

Supplementary Material
