# Author Correction: Ferromagnetic excess moments and apparent exchange bias in FeF_2_ single crystals

**DOI:** 10.1038/s41598-020-58757-2

**Published:** 2020-01-30

**Authors:** D. C. Joshi, P. Nordblad, R. Mathieu

**Affiliations:** Department of Engineering Sciences, Uppsala University, Box 534, SE-751 21 Uppsala, Sweden

Correction to: *Scientific Reports* 10.1038/s41598-019-55142-6, published online 11 December 2019

A citation was omitted in the first paragraph of this Article. Doyle *et al*. refers to Reference 1 in this Correction.
