# Supplementary material for: Ferromagnetic excess moments and apparent exchange bias in FeF2 single crystals
Source: Sci Rep. 2019 Dec 11;9:18884. doi: 10.1038/s41598-019-55142-6 (PMC6906504; doi:10.1038/s41598-019-55142-6)
Supplement: Supplementary file 1 — Supplementary Material [file 41598_2019_55142_MOESM1_ESM.pdf]

Ferromagnetic excess moments and apparent exchange bias in FeF_2 single crystals

D. C. Joshi[1*], P. Nordblad[1], R. Mathieu[1]

[1]*Department of Engineering Sciences, Uppsala University, Box 534, SE-751 21, Uppsala, Sweden*

Content

S.N.	Figure	Figure Description	Page No.
**1.**	SM1	Susceptibility in SI representation and $M_{TRM}(T)$ in μ_B/Fe of Fig. 3.	2
**2.**	SM2	M(T) under ZFC and FC conditions in magnetic field of H = 1 T for ‖c orientation.	3
**3.**	SM3	M(H) recorded at 100 K for out of plane (‖c) sample.	4
**4.**	SM4-6	M(H) recorded under FC UP and FC Down conditions from 100 K down to 35 K for three different orientations of FeF_2.	5-7
**5.**	SM7	Repetition of M(H) measurement for out of plane (‖c) sample and M(H) recorded with maximum field H=1kOe.	8
**6.**	SM8	M(H) recorded under FC in 25 Oe from 100 K down to 10, 35 and 50 K for out of plane (‖c) orientation.	9
**7.**	SM9	M(H) recorded using PPMS-VSM setup, after FC in 100 Oe from 100 K down to 5 K for out of plane (‖c) orientation.	10

Supplementary Figures:

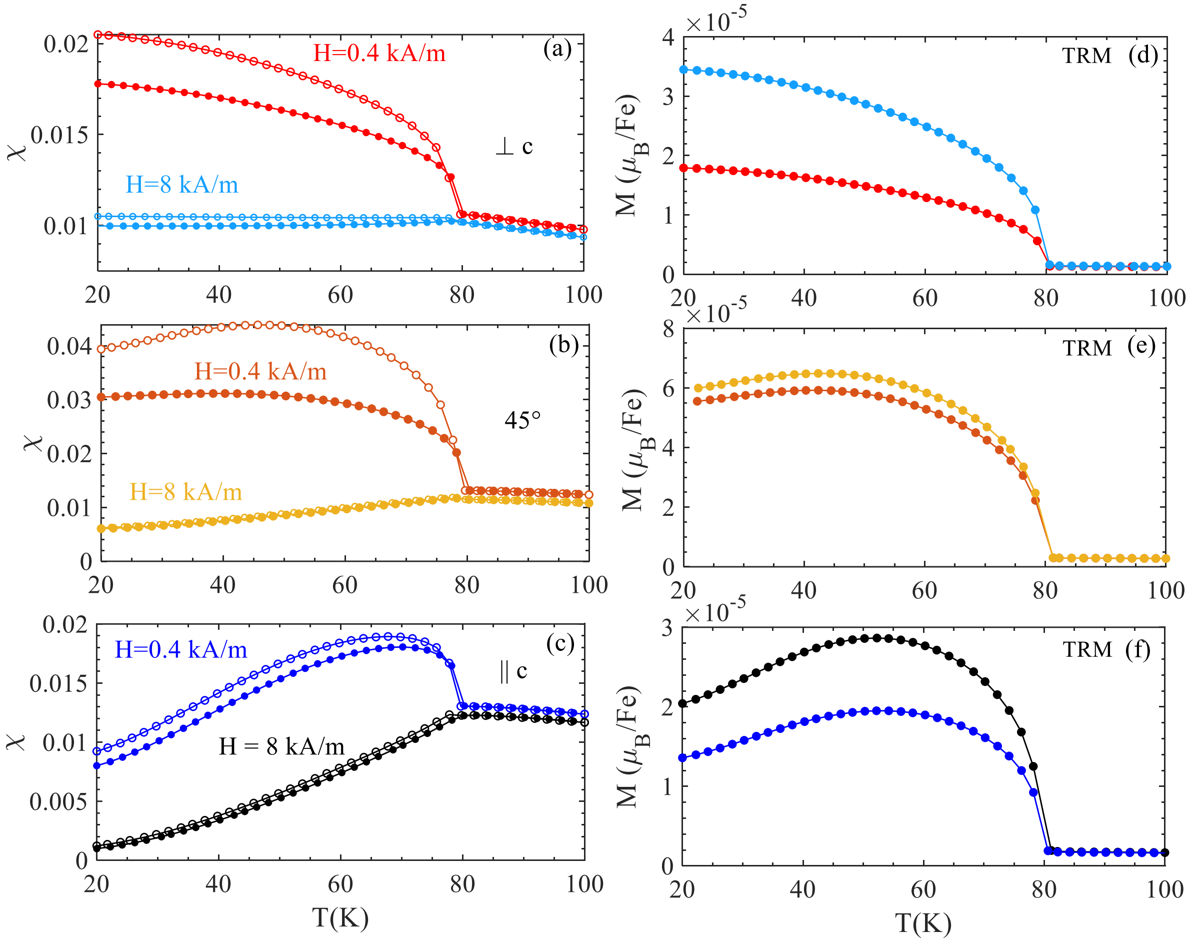

Fig. SM1: (left) ZFC/FC curves plotted as the susceptibility M/H in SI representation and $M_{TRM}(T)$ in μ_B/Fe of Fig. 3 for two selected fields H =5 Oe and 100 Oe and three orientations; (a) and (d) for ⊥ c, (b) and (e) for 45° and, (c) and (f) for parallel to *c*-axis (∥ c).

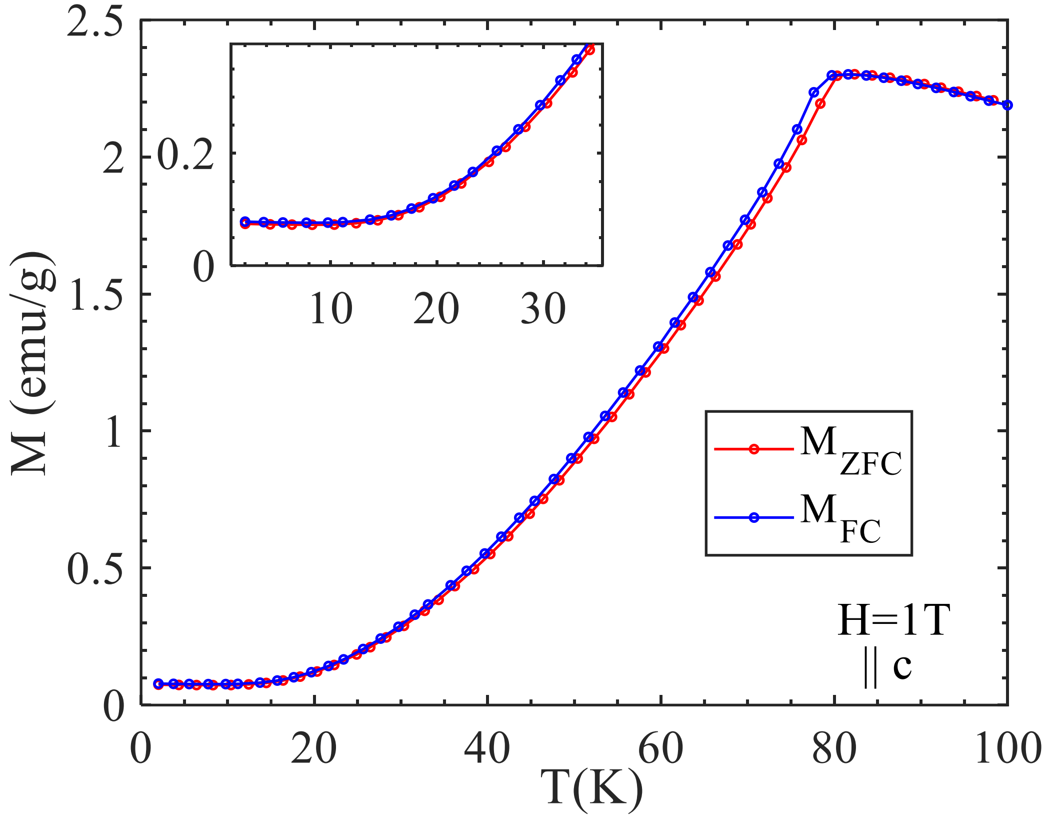

Fig. SM2: Temperature dependence of magnetization M under ZFC and FC in magnetic field of H = 1T for ||c orientation. Inset shows the zoomed view M(T) curve in the low temperature regime.

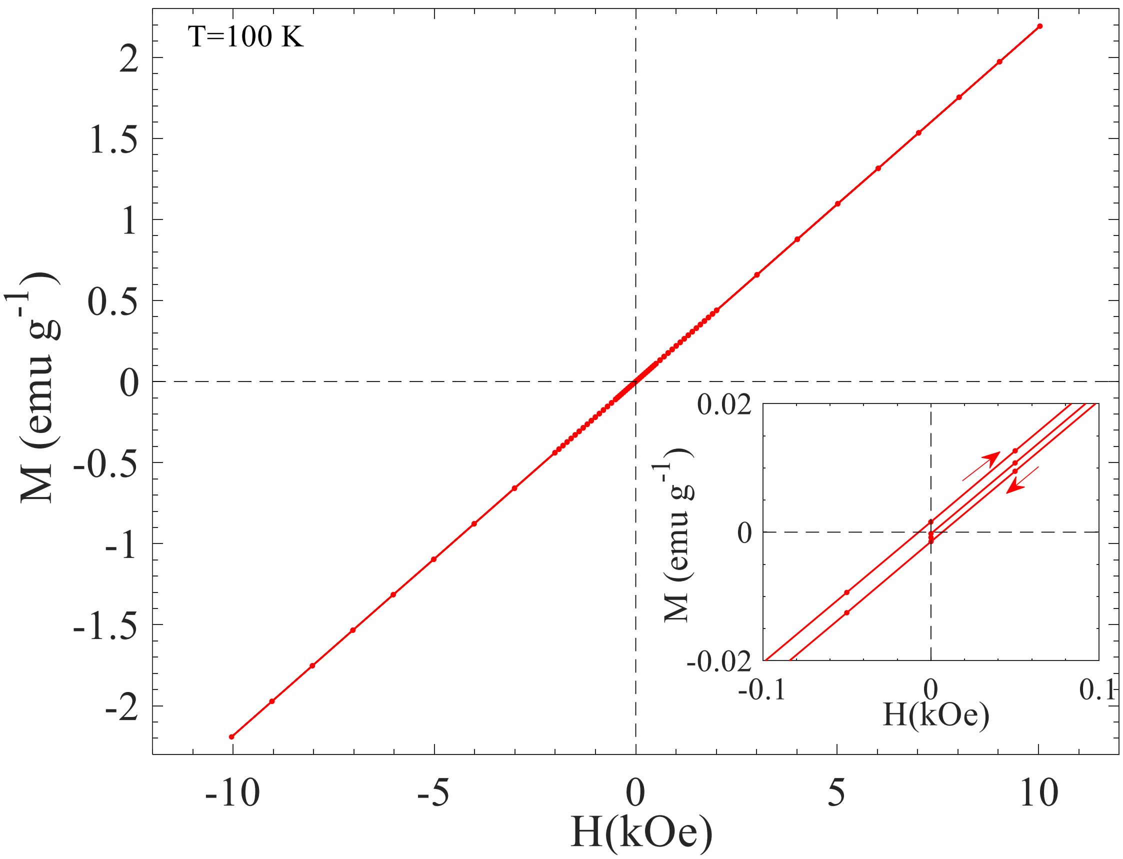

Fig. SM3: M(H) recorded with field sweep 0 Oe→+1 T→-1 T→+1 T at 100 K for out of plane (||c) sample. Inset shows the zoomed view of the main panel.

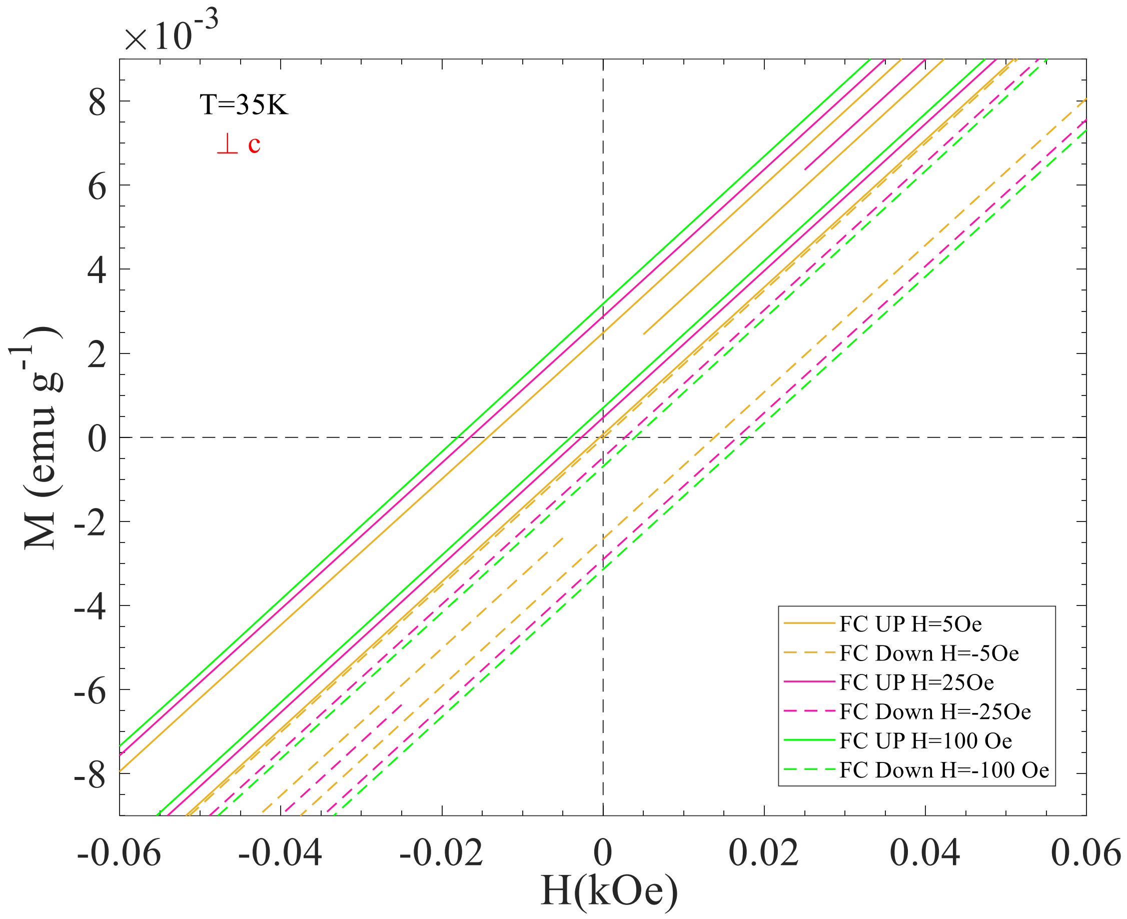

Fig. SM4: M(H) recorded under FC "UP"($+H_{FC}$→ 1 T→ -1 T→ 1 T; H_{FC}>0) and FC "Down" ($-H_{FC}$→ -1 T→ 1 T→ -1 T; $-H_{FC}$<0) conditions from 100 K down to 35 K for the in-plane (⊥ c) FeF_2 circular disk.

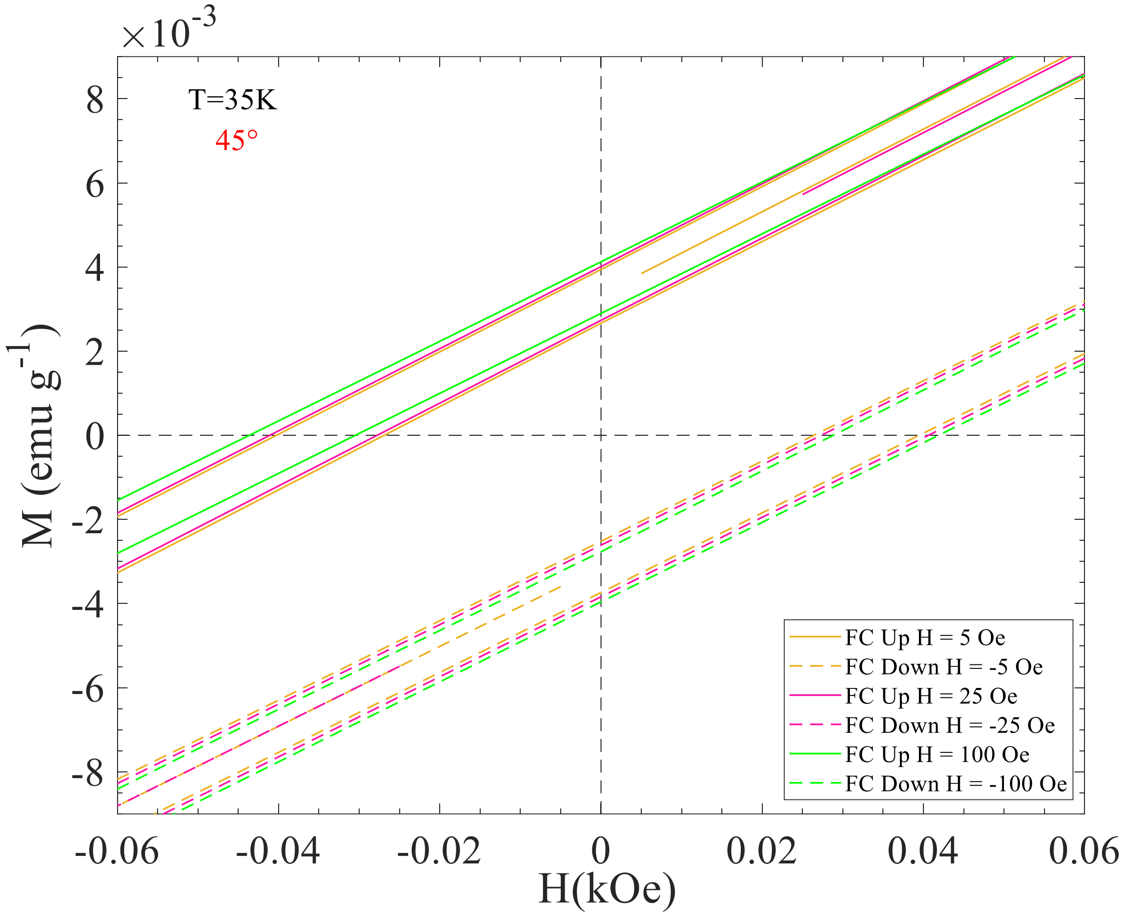

Fig. SM5: M(H) recorded under FC-UP and FC-Down conditions from 100 K down to 35 K for the 45° oriented FeF_2 circular disk.

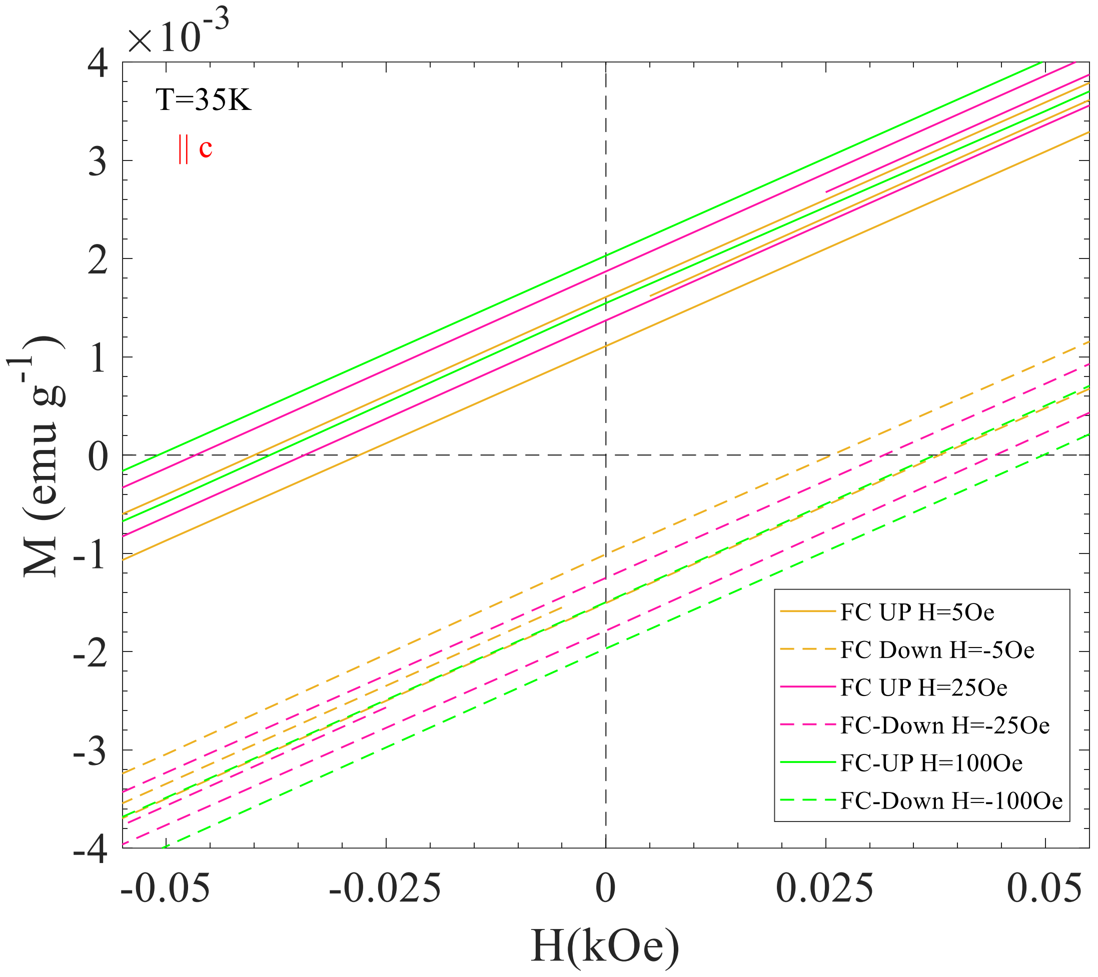

Fig. SM6: M(H) recorded under FC-UP and FC-Down conditions from 100 K down to 35 K for the out of plane (|| c) FeF_2 circular disk.

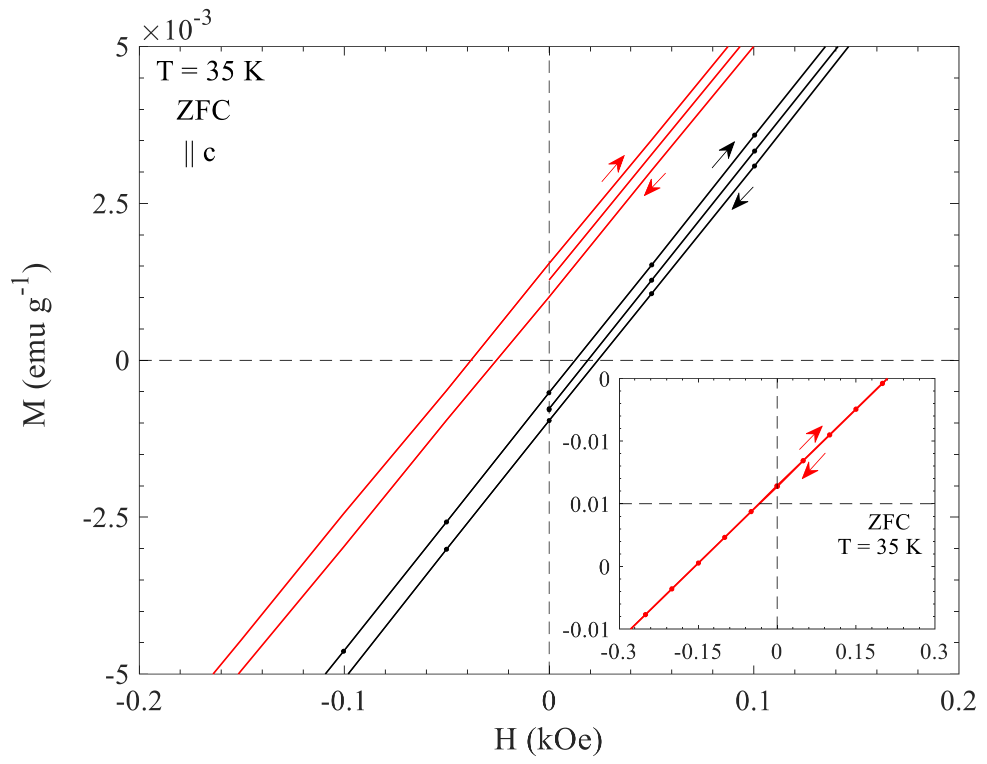

Fig SM7: M(H) measurement recorded twice with field sweep 0 → +1 T→ -1 T→ +1 T after ZFC from 100 K down to 35 K for || c. Inset: M(H) recorded with field sweep 0 → +1 kOe → -1 kOe → +1 kOe after ZFC from 100 K down to 35 K (|| c).

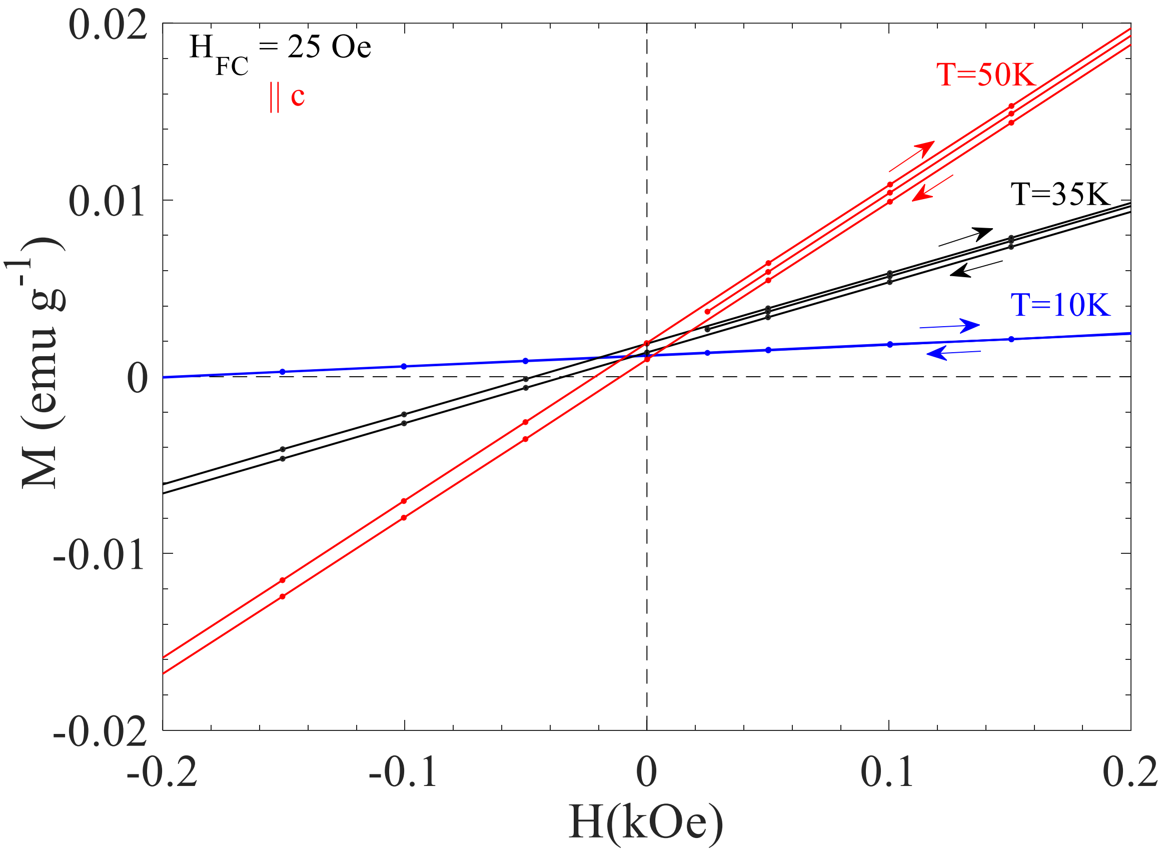

Fig. SM8: M(H) recorded with field sweep 25 Oe→+1T →-1 T→+1 T after FC in 25 Oe from 100 K down to 10, 35 and 50 K for out of plane (||c) orientation.

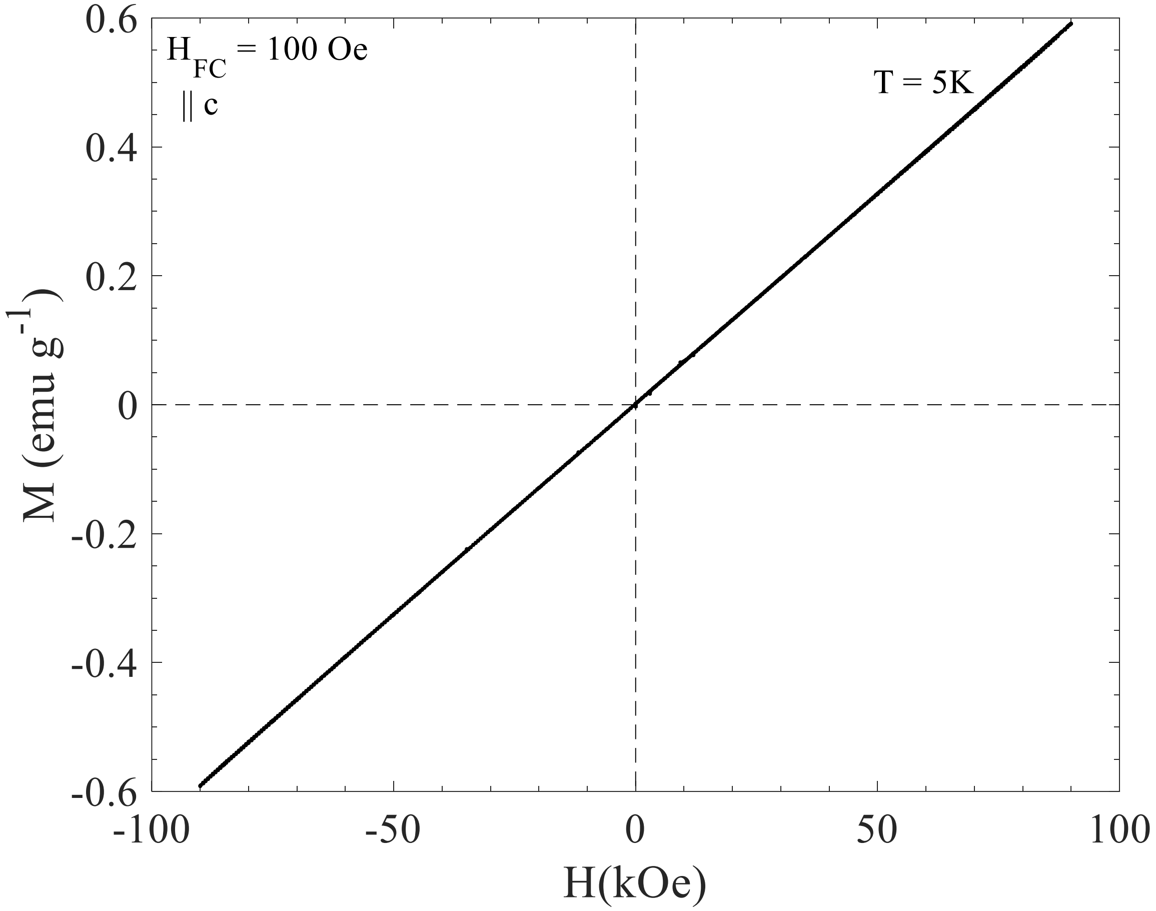

Fig. SM9: M(H) recorded with field sweep 100 Oe→+ 9 T→- 9 T→+ 9 T (using PPMS-VSM setup) after FC in 100 Oe from 100 K down to 5 K for out of plane (||c) orientation. The observed values of H_{EB} and M_R are 0.24 kOe and 1.4 × 10^{-3} emu/g, which are consistent with the values of Fig 5.
